# Chemical diversity and antifouling activity of geniculate calcareous algae (Corallinales, Rhodophyta) from Brazil

**DOI:** 10.7717/peerj.15731

**Published:** 2023-08-14

**Authors:** Ellen A. de S. Oliveira, Juliana de A.S. Oliveira, Priscila R. Araújo, Frederico T.S. Tâmega, Ricardo Coutinho, Angelica R. Soares

**Affiliations:** 1 Programa de Pós-Graduação em Biotecnologia Marinha, IEAPM/ Universidade Federal Fluminense (UFF), Arraial do Cabo, Rio de Janeiro, Brazil; 2 Departamento de Biotecnologia Marinha, Instituto de Estudos do Mar Almirante Paulo Moreira, Arraial do Cabo, Rio de Janeiro, Brazil; 3 Grupo de Produtos Naturais de Organismos Aquáticos, Universidade Federal do Rio de Janeiro (NUPEM), Macaé, Rio de Janeiro, Brazil; 4 Department of Environmental Chemistry, Swiss Federal Institute of Aquatic Science and Technology (EAWAG), Dübendorf, Switzerland

**Keywords:** Geniculate calcareous algae, Crude extract, Chemical composition, Metabolomics, Multivariate analysis, Biofouling

## Abstract

Marine biofouling is a natural process by which many organisms colonize and grow in submerged structures, causing serious economic consequences for the maritime industry. Geniculate calcareous algae (GCA; Corallinales, Rhodophyta) produce bioactive secondary metabolites and are a promise for new antifouling compounds. Here, we investigated the antifouling activity of four GCA species—*Amphiroa beauvoisii*, *Jania sagittata* (formerly *Cheilosporum sagittatum*), *Jania crassa*, and *Jania prolifera* (formerly *Amphiroa flabellata*)—from the Brazilian coast against macro- and microorganisms. Simultaneously, metabolomic tools were applied to assess the chemical profiles of these seaweeds using gas chromatography coupled to mass spectrometry (GC-MS). Data analysis by principal component and molecular networking analyses used the global natural products social molecular networking platform (GNPS). Our results showed that all extracts were active against different strains of marine bacteria and that the *J*.* sagittata* (JsSI) extract showed the highest percentage of bacterial inhibition. The *J. sagittata* (JsSI) extract was the most active against the mussel *Perna perna*, showing 100% byssus inhibition. Regarding toxicity, only the *J. crassa* (JcP) extract showed a 20% mortality rate. The chemical profiles of the evaluated GCA extracts differed qualitatively and quantitatively. Yet, the steroid (3*β*)-cholest-5-en-3-ol was the major compound commonly identified in all extracts, with the exception of *J. sagittata* (JsSI). Moreover, we observed intra- and interspecific chemical variabilities among GCA extracts for the different populations, which could explain their antifouling activity variability. This study contributed new information about the chemical compounds produced by this group of seaweeds and showed its antifouling potential. These GCA species may be the subject of future studies to obtain new bioactive compounds with biotechnological potential in maritime areas.

## Introduction

Marine biofouling is the process of colonization and growth of sessile organisms on submerged surfaces such as ship hulls, platforms, pipes, and buoys (Maréchal & Hellio, 2009). After the adsorption of organic particles on these surfaces, the formation of bacterial biofilm occurs, enabling the proliferation of microorganisms (Wahl, 1989; Dang et al., 2007; Martín-Rodríguez et al., 2015). Subsequently, this biofilm may also facilitate the colonization and growth of macroorganisms such as mussels, seaweeds, barnacles, and bryozoans (Wahl, 1989; Martín-Rodríguez et al., 2015). The development of micro- and macrofouling seriously impacts the marine industry worldwide as it affects the efficiency of maritime transport due to the greater roughness and corrosion of its vessels (Ali et al., 2020; Ferreira et al., 2020), increasing maintenance costs (Cao et al., 2011) and fuel consumption (Ali et al., 2020). Furthermore, marine biofouling is one of the main vectors of introduction of exotic/invasive species (Davidson et al., 2016; Vimala, 2016; Ali et al., 2020).

The main strategy to minimize the impacts of biofouling in the shipbuilding industry focused on the use of biocides containing arsenic, mercury, lead, and tributyltin (TBT) to cease or inhibit the colonization of fouling organisms (Ali et al., 2020). However, these biocides showed a high level of environmental contamination and posed risks to marine organisms (Silva et al., 2018; Ali et al., 2020; Han et al., 2021). Therefore, after proving the high toxicity of tributyltin to target and non-target marine species, its application in the coating of ships as a biocide was banned in 2008 (Batista-Andrade et al., 2018). Other biocides with less toxic formulations are now used to replace tributyltin in the control of biofouling, such as Diuron, Irgarol 1051, and Sea-Nine 211, for example. However, studies with these compounds alone or in mixtures still report their negative effects on several marine organisms (Wang et al., 2011; Batista-Andrade et al., 2018).

Seaweeds are both a rich source of bioactive compounds and the target of biotechnological studies. In the natural environment, they produce a variety of chemical compounds, known as secondary metabolites or natural products, capable of preventing the growth of epibiont organisms (Da Gama et al., 2008; Da Gama, Plouguerné & Pereira, 2014; Othmani et al., 2015; Qian et al., 2015; Carvalho et al., 2016; Sánchez-Lozano et al., 2019). In this context, the use of these natural compounds could be seen as an efficient alternative for natural biocides with antifouling potential. The production of these metabolites is known to change according to the influence of several factors, such as temperature (Sudatti et al., 2011), location (Plouguerné et al., 2010; Stengel, Connan & Popper, 2011), season (Stengel, Connan & Popper, 2011; Mansur, Brown & Billington, 2020), and exposure to ecological interactions (Stengel, Connan & Popper, 2011). Geniculate calcareous algae (GCA; Corallinales, Rhodophyta) are included in this group of seaweeds, whose thalli consist of alternating calcified and noncalcified segments, unlike non-geniculate calcareous algae, which have entirely calcified thalli (Johansen, 1981). Both calcareous algae are distributed worldwide (Foster, 2001; Harvey et al., 2005; Riosmena-Rodríguez, Nelson & Aguirre, 2017) and distinguish themselves from other red algae by the presence of calcium carbonate (CaCO_3_) in their cellular walls in the form of calcite (Nash & Adey, 2017).

GCA produce a variety of chemical compounds, such as fatty acids (Cikoš et al., 2021), sterols (Caf et al., 2019), and hydrocarbons (Ahmed et al., 2011). Tannins, flavonoids, alkaloids, and carotenoids (Akbary, Adeshina & Jahanbakhshi, 2020; Cikoš et al., 2021) have also been found in the chemical profile of these seaweeds. These compounds are responsible for different biological activities (Raj et al., 2019; Cikoš et al., 2021; Righini et al., 2021; Mofeed et al., 2022), including antifouling (Medeiros, Gama & Gallerani, 2007; Kantida, Asha & Sujatha, 2012; Deepa, Srikumar & Padmakumar, 2014). Nevertheless, this group of seaweed is numerous and widely distributed. Therefore, the number of studies focused on exploring and understanding the chemical composition and biotechnological applications of GCA remains limited.

Considering the importance of the bioactive compounds geniculate calcareous algae produce and the scarce investigation of them on the Brazilian coast, this study aimed to analyze the intra- and interspecific chemical profile and antifouling potential of the crude extracts of four GCA species—*Amphiroa beauvoisii* J.V. Lamouroux, *Jania sagittata* (J.V. Lamouroux) Blainville (formerly *Cheilosporum sagittatum* (J.V. Lamouroux) Areschoug), *Jania crassa* J.V. Lamouroux, and *Jania prolifera* A.B. Joly (formerly *Amphiroa flabellata* Harvey)—collected in Arraial do Cabo, Rio de Janeiro, Brazil.

## Materials & Methods

### Study area and sampling sites

**Figure 1:**
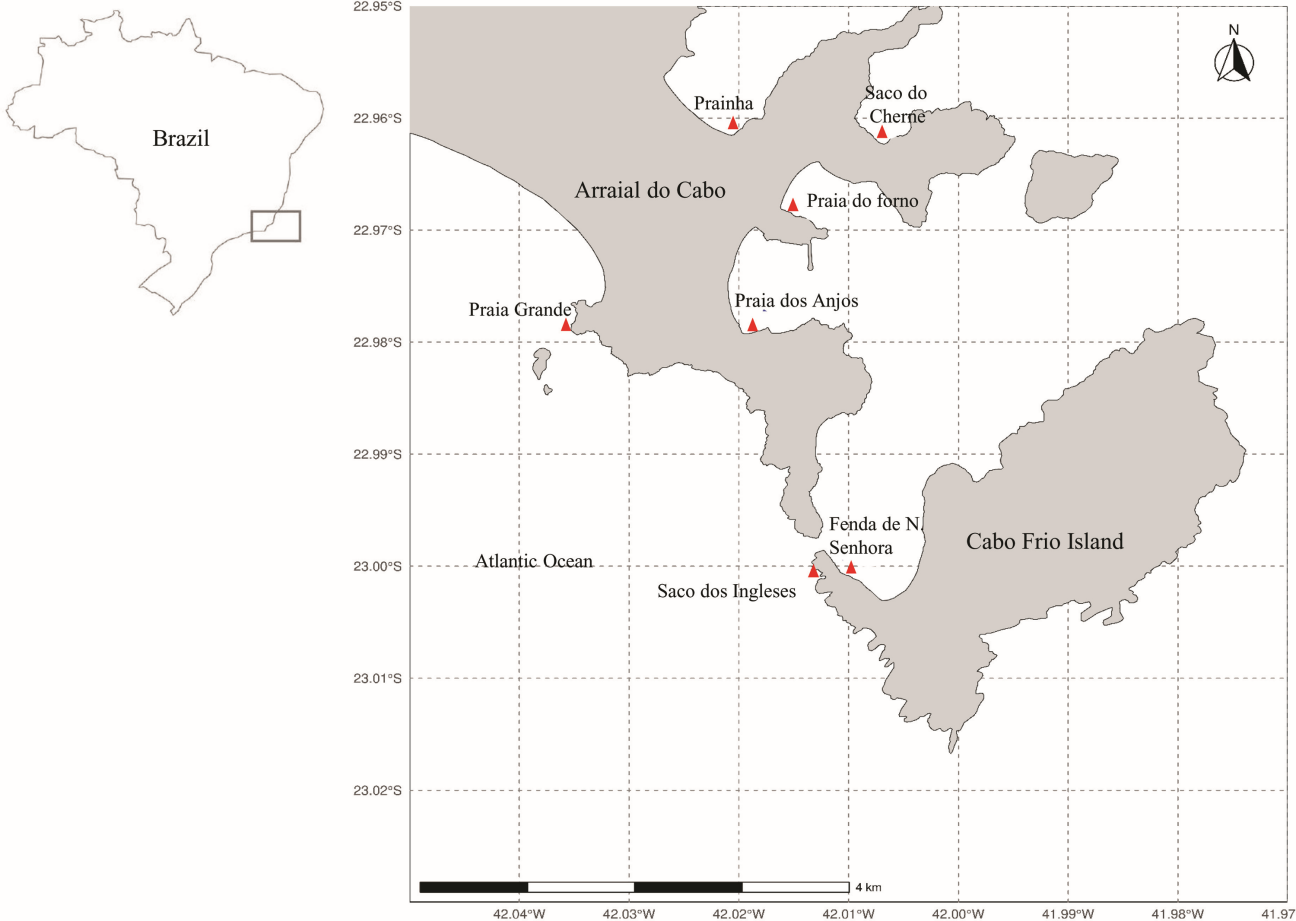
Map of Arraial do Cabo (Rio de Janeiro, Brazil) indicating the sampling sites (red): Fenda de Nossa Senhora (FNS), Prainha (P), Praia do Forno (PF), Saco do Cherne - rocky shore (SCC), Saco do Cherne - articuliths bed (SCB), Praia dos Anjos, Saco dos ingleses (SI) and Praia Grande (PG).

Arraial do Cabo is on the coast of the state of Rio de Janeiro, in Southeastern Brazil (Fig. 1). The region is influenced by local summer and spring upwellings associated with the local wind regime and bathymetry (Castelão, 2012; Belem, Castelao & Albuquerque, 2013). Although these upwellings bring cold nutrient-rich waters to the surface, average sea surface temperatures at the Arraial do Cabo Bay remain predominantly above 20 °C (Guimaraens & Coutinho, 1996; Candella, 2009). Thus, while the surrounding rocky shores are largely characterized by tropical reef communities, Arraial do Cabo represents a unique site with the co-occurrence of both tropical and subtropical marine species (Laborel, 1970; Lanari & Coutinho, 2014).

The seaweed samples were manually collected in several intertidal and infralittoral regions in Arraial do Cabo by Scuba diving (Fig. 1) in the summer of 2018. Study sites included Fenda de Nossa Senhora, Prainha, Praia do Forno, Saco do Cherne (with a rocky shore and articulith beds, Tâmega et al., 2017; Tâmega et al., 2021), Praia dos Anjos, Saco dos Ingleses, and Praia Grande. A total of nine samples were collected, five of *A. beauvoisii*, two of *J. crassa*, one of *J. sagittata*, and one of *J. prolifera*. Subsequently, they were washed (in seawater to remove sand and associated organisms from them), frozen, and lyophilized. These seaweed samples are housed in the scientific collection at Instituto de Estudos do Mar Almirante Paulo Moreira (IEAPM, Table 1).

**Table 1:** Species of GCA sampled and their collection sites.

**Specie**	**Code**	**Collection site**	**Wet weight (g)**	**Voucher number**
*Amphiroa beauvoisii*	AbFNS	Fenda de Nossa Senhora	400.00	3372
*Amphiroa beauvoisii*	AbP	Prainha	474.40	3373
*Amphiroa beauvoisii*	AbPF	Praia do Forno	184.95	3374
*Amphiroa beauvoisii*	AbSCB	Saco do Cherne (articulith beds)	192.24	3375
*Amphiroa beauvoisii*	AbSCC	Saco do Cherne (rocky shore)	265.53	3376
*Jania sagittata*	JsSI	Saco dos Ingleses	207.48	3377
*Jania crassa*	JcPA	Praia dos Anjos	213.37	3378
*Jania crassa*	JcP	Prainha	309.11	3379
*Jania prolifera*	JpPG	Praia Grande	117.45	3380

### Preparation of seaweed extracts

The dried frozen seaweeds were extracted in a mixture of ethyl acetate and methanol (EtOAc: MeOH 1:1 v/v) in a proportion of 3.5 mL of solution for every 1g of sample (dry weight). The extraction was carried out with a standing time of 2 h/16 h/2 h, respectively, with the assistance of ultrasound. Subsequently, the extracts were filtered by gravity and concentrated under reduced pressure.

### Extract chemical profile

The obtained extracts were analyzed by gas chromatography coupled to mass spectrometry (GC-MS) to assess the chemical diversity of GCA. Before analysis, the extracts were diluted in dichloromethane (HPLC, Tedia) and filtered through 0.45-µm PTFE filters (Millipore; EUA) to remove any insoluble constituents. Subsequently, the solvent was evaporated, the samples were lyophilized, and the remaining material was resuspended in ethyl acetate (HPLC grade; Tedia) to a final concentration of 1 mg/mL. GC-MS analysis was carried out using Shimadzu CG-2010 equipment coupled to a QP-2010 ultra-mass spectrometer comprising an AOC-20i auto-injector and a 30-m × Φ int 0.25-mm Rtx-1MS column.

A 1.20 mL/min column flow rate was used in split mode with a ratio of 1/5 and helium as carrier gas. The injector temperature was kept at 280 °C and the column was heated to 150 °C for 3 min, followed by a temperature ramp to 300 °C (rate of 6 °C/min), and maintained at 300 °C for 5 min, totaling 33 min. A mass detector was used in electron impact mode (70 eV) with an interface temperature at 300 °C and an ion source at 200 °C. Chemical profiles were analyzed based on their mass spectra and retention time. Chemical compounds were identified by comparing their mass spectra with those available in the NIST 11 library, considering similarity indices equal to or greater than 85% (SI ≥ 85%).

The chromatograms obtained by GC-MS were converted into a computable document format (CDF) and uploaded into the global natural products social molecular networking (GNPS) platform (Wang et al., 2016). A metadata table was created to help us to observe and identify possible patterns or groupings between them. Results were processed on the Cytoscape software for networking visualization, enabling us to identify similar chemical classes/molecular families between the studied GCA extracts. A cosine value ≥ 0.7 was considered in the identification of compounds. The data obtained on this platform were compared to the NIST 11 library.

### Antibacterial activity

Antibacterial bioassays followed the methodology described in Devi et al. (2011) with modifications. The antibacterial activity of all extracts was tested at their natural concentration and evaluated against bacterial strains associated with marine fouling: four strains of Gram-negative marine bacteria (*Polaribacter irgensii*, *Pseudoalteromonas elyakovii*, *Pseudomonas fluorescens*, and *Vibrio aestuarianus*) and a strain of Gram-positive marine bacteria (*Shewanella putrefaciens*). Antibacterial tests were performed by disc diffusion assay (*n* = 5) at an optical density (O.D) of 1.5–1.8 at 630 nm. The extracts were solubilized in ethyl acetate and methanol (1:1 v/v) and applied to sterile discs (five mm in diameter) made of filter paper (Whatman no. 1) (Table 2). Disks with the antibiotic streptomycin (Sigma-Aldrich, St. Louis, MO, USA) were used as a positive control (*n* = 5) at a concentration of 10 mg/g. After a 24-h incubation at 30 °C, the diameter (mm) of the inhibition halo around the disks was measured using the ImageJ software (version 1.52a).

**Table 2:** Natural concentration of GCA extracts applied in the antifouling and antibacterial activities.

**Sample**	**Natural** **concentration (mg/g)**
AbFNS	38
AbP	31
AbPF	39
AbSCB	21
AbSCC	28
JsSI	26
JcPA	24
JcP	37
JpPG	21

### Antifouling activity against the mussel *Perna perna*

The assay of antifouling activity against the mussel *Perna perna* was modified based on the method described by Da Gama et al. (2003). Specimens were collected in the coastal area of Praia Grande (Arraial do Cabo, Brazil), and carefully separated and cleaned. Individuals were selected if they met three criteria: shell length between 1.6–2.0 cm, active exposure of their feet, and capability to crawl.

The extracts were solubilized in ethyl acetate and methanol (1:1 v/v) at their natural concentration (Table 2) and incorporated into filter paper discs (five cm, *n* = 10). After the solvent was completely evaporated, the discs were placed at the bottom of glass Petri dishes (60 × 15 mm). Discs soaked only in seawater were used as a null control. After 24 h, the number of byssus fixed by the mussels in each experimental condition was evaluated. At the end of the tests, the mussels were placed in containers with filtered seawater. After this period, the response of the individuals to touch and the observation of tissue loss and open valve were followed to measure the toxic effect of the extracts.

### Data analysis

Multivariate analysis was performed to investigate the possible chemical profile variability of GCA extracts. The Correlation Warping Algorithm was used to correct the baseline of each chromatogram and peak retention time (Nielsen, Carstensen & Smedsgaard, 1998). A matrix with all aligned chromatograms was constructed and used for Principal Component Analysis (PCA) using the Rstudio software and environment (http://www.R-project.org) and the “ChemometricsWithR” package (Wehrens, 2011). Activity against marine bacteria was expressed in millimeters (mm), whereas the values of *P. perna* byssus fixed on the plates, in percentages.

For the experiment with *P. perna*, the values of byssus fixed on the plates were converted into percentages for further analysis. The assumptions of normality and homogeneity required for ANOVA were evaluated using the Shapiro–Wilk and Cochran C tests, respectively. One-way ANOVA was used for the antibacterial experiment to compare inhibition halo values between control (positive) and treatments (extracts) for the same bacteria. For the *P. perna* experiment, the comparison was established between the percentage of byssus fixed between control (negative) and treatments (extracts). The post-hoc Tukey’s test was used to assess significant differences (*p* < 0.05). These analyses were performed using the Statistica 8 software (Statsoft, Inc., Tulsa, OK, USA).

## Results

### Extract chemical profile

**Figure 2:**
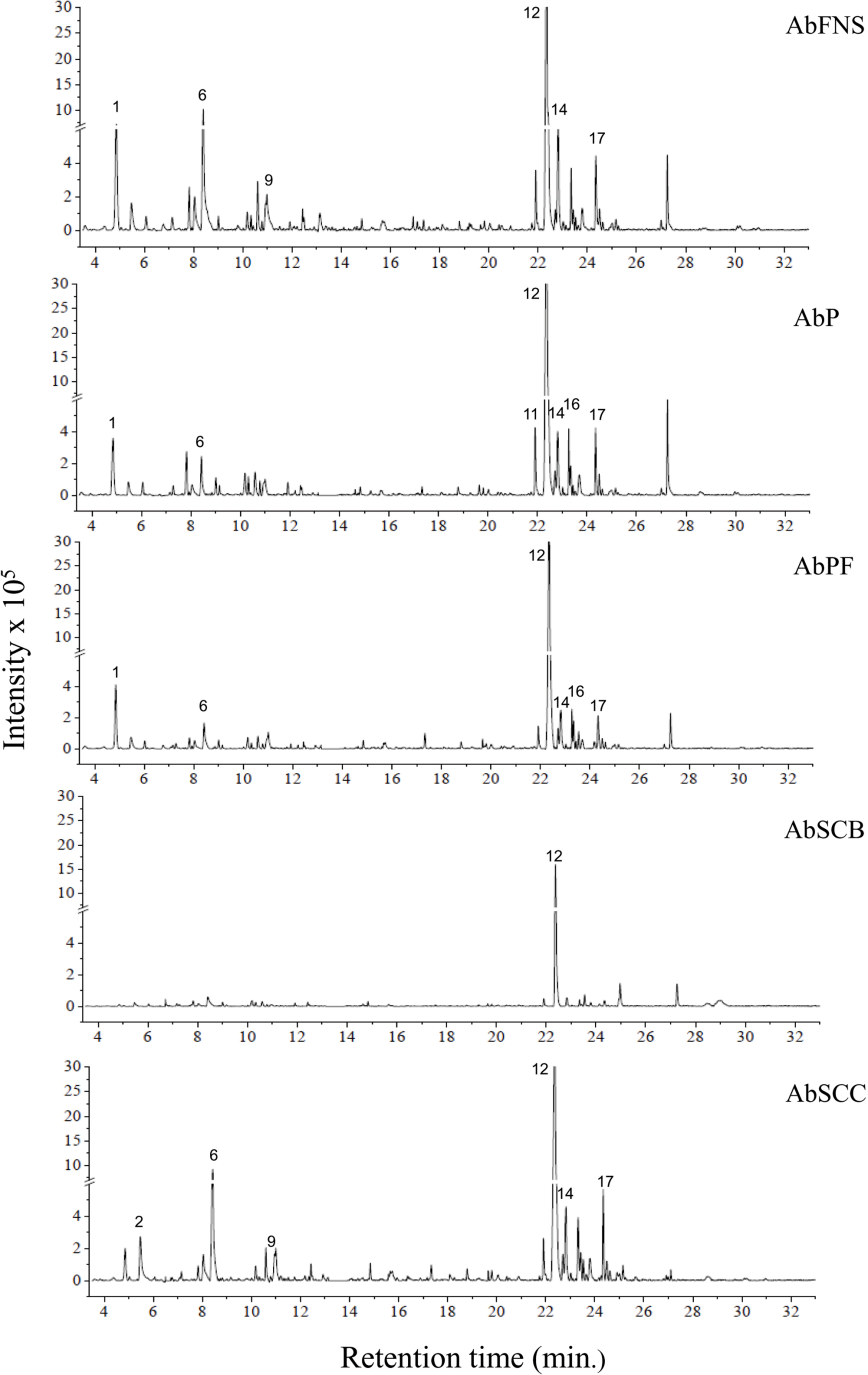
Chromatograms of GCA extracts obtained by GC-MS. AbFNS (*A. beauvoisii*—Fenda de Nossa Senhora); AbP (*A. beauvoisii*—Prainha); AbPF (*A. beauvoisii*—Praia do Forno); AbSCB (*A. beauvoisii*—Saco do Cherne—articuliths bed) and AbSCC (*A. beauvoisii*—Saco do Cherne—rocky shore). Numbers indicate the most abundant compouds (relative area ≥ 2%).

**Figure 3:**
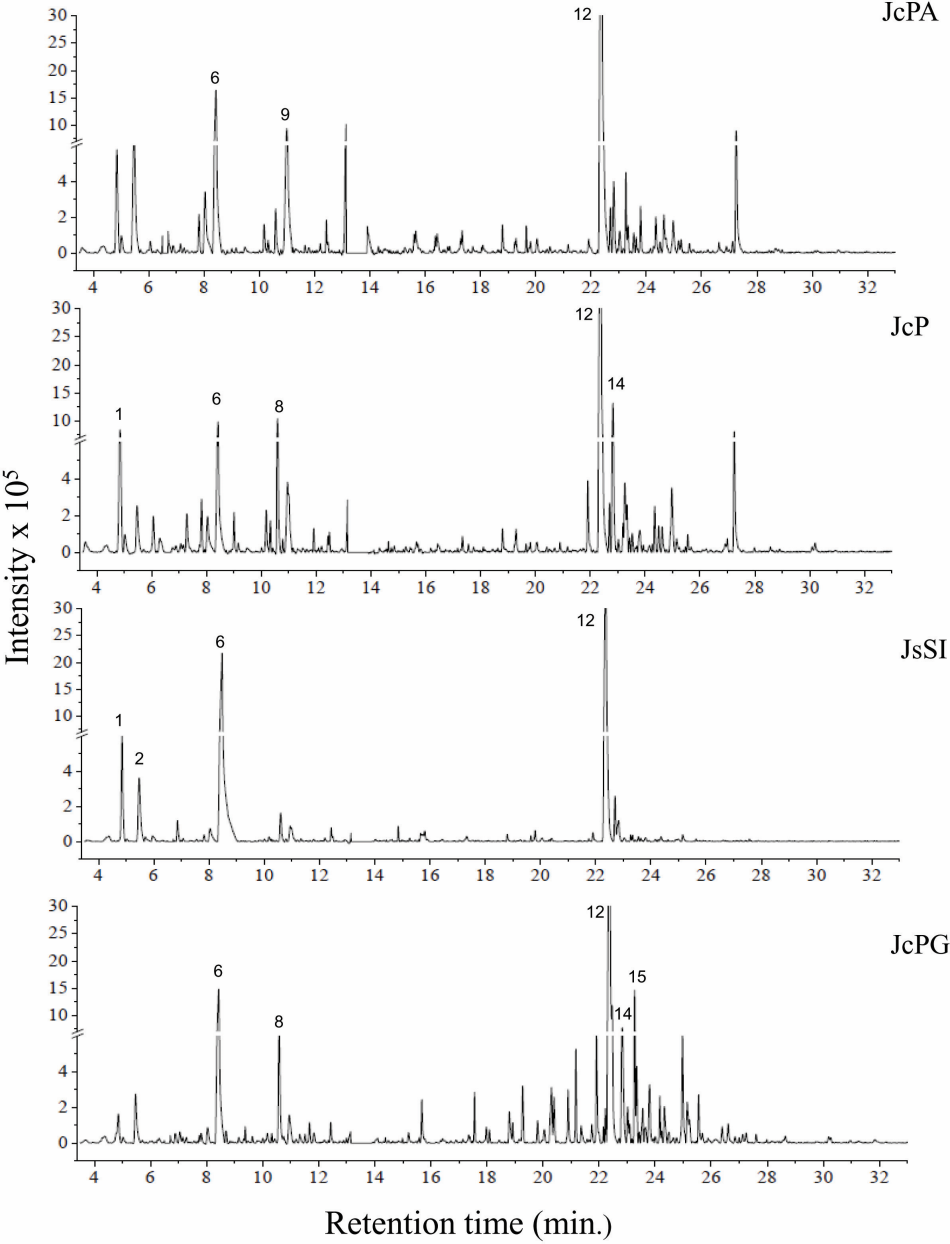
Chromatograms of GCA extracts obtained by GC-MS. JcPA (*J. crassa*—Praia dos Anjos); JcP (*J. crassa*—Prainha); JsSI (*J. sagittata*—Saco dos Ingleses) and JpPG (*J. prolifera*—Praia Grande). Numbers indicate the most abundant compouds (relative area ≥ 2%).

**Figure 4:**
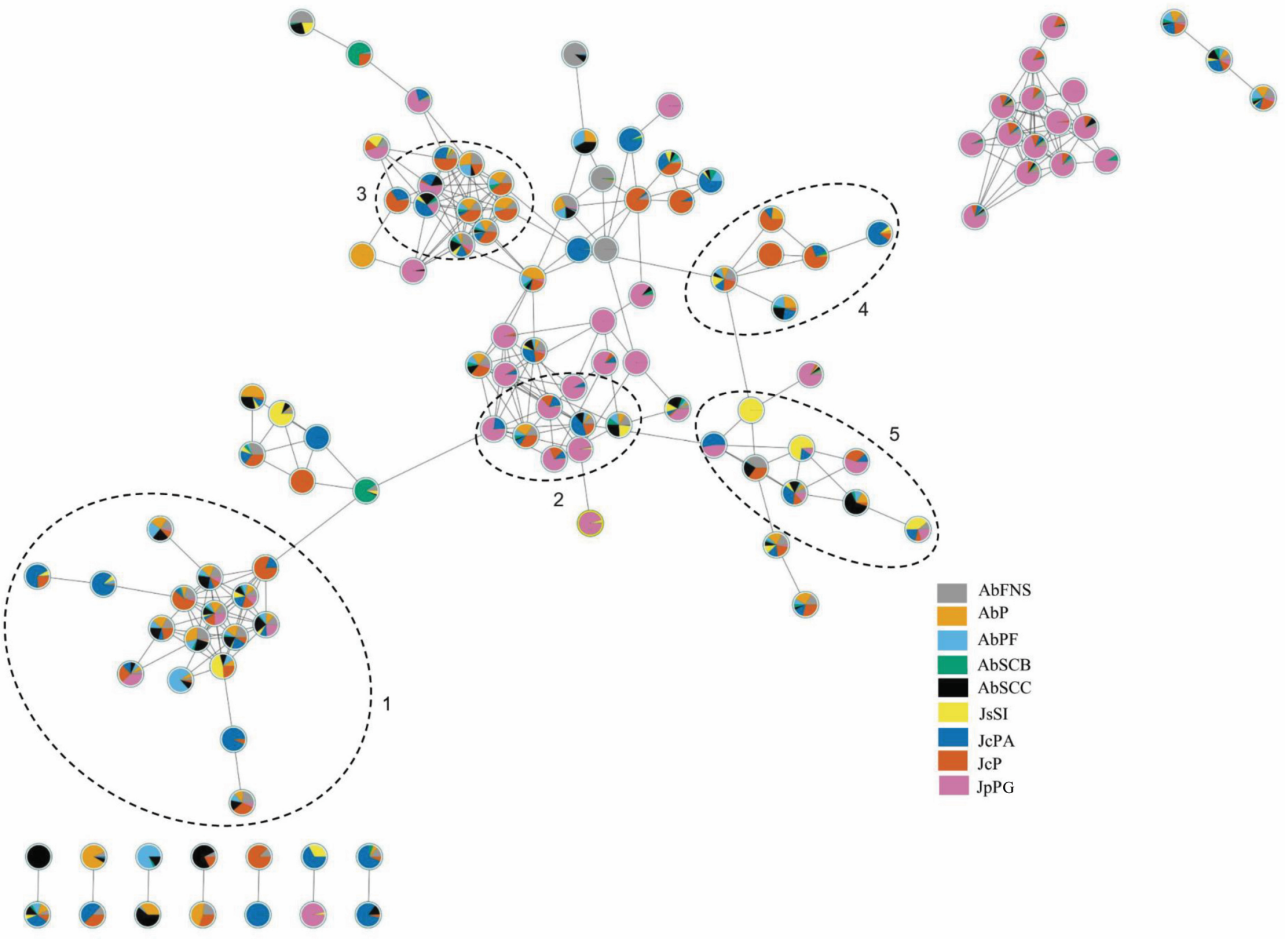
Molecular network obtained on the GNPS platform from extracts of GCA. Each number represents a different molecular family: sterols (1), fatty acid esters (2), fatty alcohols (3), hydrocarbons (4) and fatty acids (5). AbFNS (*A. beauvoisii*—Fenda de Nossa Senhora); AbP (*A. beauvoisii*—Prainha); AbPF (*A. beauvoisii*—Praia do Forno); AbSCB (*A. beauvoisii*—Saco do Cherne, articuliths bed); AbSCC (*A. beauvoisii*—Saco do Cherne costão); JcPA (*J. crassa*—Praia dos Anjos); JcP (*J. crassa*—Prainha); JsSI (*J. sagittata*—Saco dos Ingleses) and JpPG (*J. prolifera*—Praia Grande).

The chromatograms obtained by GC-MS analysis showed both qualitative and quantitative intra- and interspecific chemical variability in the analyzed extracts (Figs. 2–3). We observed more complex chemical profiles in the *J. crassa* (JcP) and *J. prolifera* (JpPG) extracts, whereas we found a simpler composition in *A. beauvoisii* (AbSCB) and *J. sagittata* (JsSI) extracts. The molecular network created on the GNPS platform also evinced this variability by grouping similar classes of metabolites, such as sterols (1), fatty acid esters (2), fatty alcohols (3), hydrocarbons (4), and fatty acids (5) (Fig. 4). A total of 17 compounds in GCA extracts, were identified through the mass spectra of the GNPS platform and NIST, 11 (Table 3).

**Table 3:** Compounds from GCA extracts annotated by GC-MS and identified by NIST library (≥ 85%) and/or GNPS platform (cosine ≥ 0.7). X: compounds identified by both database (NIST and GNPS); Y: compounds identified only in the NIST database.

**Peak**	**tR (min** ***)***	**Phytochemical components**	**Molecular formula**	**NIST**	**GNPS**	**Relative area (%)**								
						**AbFNS**	**AbP**	**AbPF**	**AbSCB**	**AbSCC**	**JsSI**	**JcP**	**JcPA**	**JpPG**
1	4.86	Heptadecane	C_17_H_36_	X	X	4.45	3.58	5.38	0.30	1.41	4.61	2.36	1.05	0.76
2	5.47	Tetradecanoic acid	C_14_H_28_O_2_	X	X	1.13	0.82	0.99	0.55	2.17	3.21	0.79	1.60	1.41
3	6.91	Pentadecanoic acid	C_15_H_30_O_2_	Y	–	0.60	–	0.43	–	–	1.2	0.33	0.07	0.39
4	7.82	Hexadecanoic acid methyl ester	C_17_H_34_O_2_	X	X	0.90	1.50	0.52	0.69	0.39	0.17	0.58	0.23	0.15
5	8.04	Palmitoleic acid	C_16_H_30_O_2_	X	X	1.84	0.47	0.48	0.43	1.87	0.89	0.87	1.09	0.63
6	8.43	Palmitic acid	C_16_H_32_O_2_	X	X	7.93	2.20	2.13	1.90	8.05	31.35	3.25	4.36	9.03
7	10.20	(Z.Z)-9.12-Octadecadien-1-ol	C_18_H_34_O_2_	X	X	0.44	0.94	0.77	1.08	0.52	0.13	0.50	0.21	0.33
8	10.59	Phytol	C_20_H_40_O	Y	–	1.22	1.02	0.80	0.69	1.02	0.91	2.00	0.53	2.00
9	11.00	Oleic acid	C_18_H_34_O_2_	X	X	2.28	1.78	1.94	–	2.54	1.02	1.59	5.14	1.41
10	21.18	(Z)-7-Hexadecenal	C_16_H_30_O	X	X	–	–	–	–	–	–	0.04	0.04	1.33
11	21.91	((3 *β*)cholesta-5.22-dien-3-ol	C_27_H_44_O	X	X	1.26	2.22	1.24	0.88	1.13	0.23	0.69	0.09	1.66
12	22.36	(3 *β*) cholest-5-en-3-ol	C_27_H_46_O	X	X	21.70	37.11	39.07	35.84	28.2	27.53	16.62	12.63	19. 49
13	22.70	Desmosterol	C_27_H_44_O	X	X	0.81	0.75	1.00	–	0.51	1.56	0.37	0.41	0.05
14	22.82	(3 *β*. 5 *α*)-ergosta-7-en-3-ol	C_27_H_46_O	X	X	4.24	3.08	2.97	1.76	3.51	1.22	3.15	0.52	3.08
15	23.27	Oleic anhydride	C_36_H_66_O_3_	X	X	0.16	0.03	0.13	–	0.12	0.32	0.92	0.45	3.86
16	23.34	Stigmasterol	C_29_H_48_O	X	X	1.57	2.25	2.13	0.83	1.78	0.26	0.56	0.18	1.37
17	24.35	Sitosterol	C_29_H_50_O	X	X	2.04	2.53	2.03	0.99	3.14	0.06	0.59	0.28	1.06

We indicated compounds with a relative area equal to or greater than 2% (of each extract) in their respective chromatograms (Figs. 2–3). The steroid (3 *β*)-cholest-5-en-3-ol (peak 12) was the most abundant compound in all samples of *A. beauvoisii* (AbFNS 21.70%), (AbP 37.11%), (AbPF 39.07%), (AbSCB 35.84%) and (AbSCC 28.20%); in both samples of *J. crassa* (JcP 16.62%) and (JcPA 12.63%); and that of *J. prolifera* (JpPG 19.49%). The *J. sagittata* (JsSI) extract, however, showed palmitic acid (peak 6) as its most abundant compound, with a 31.35% relative area.

### Principal component analysis (PCA)

**Figure 5:**
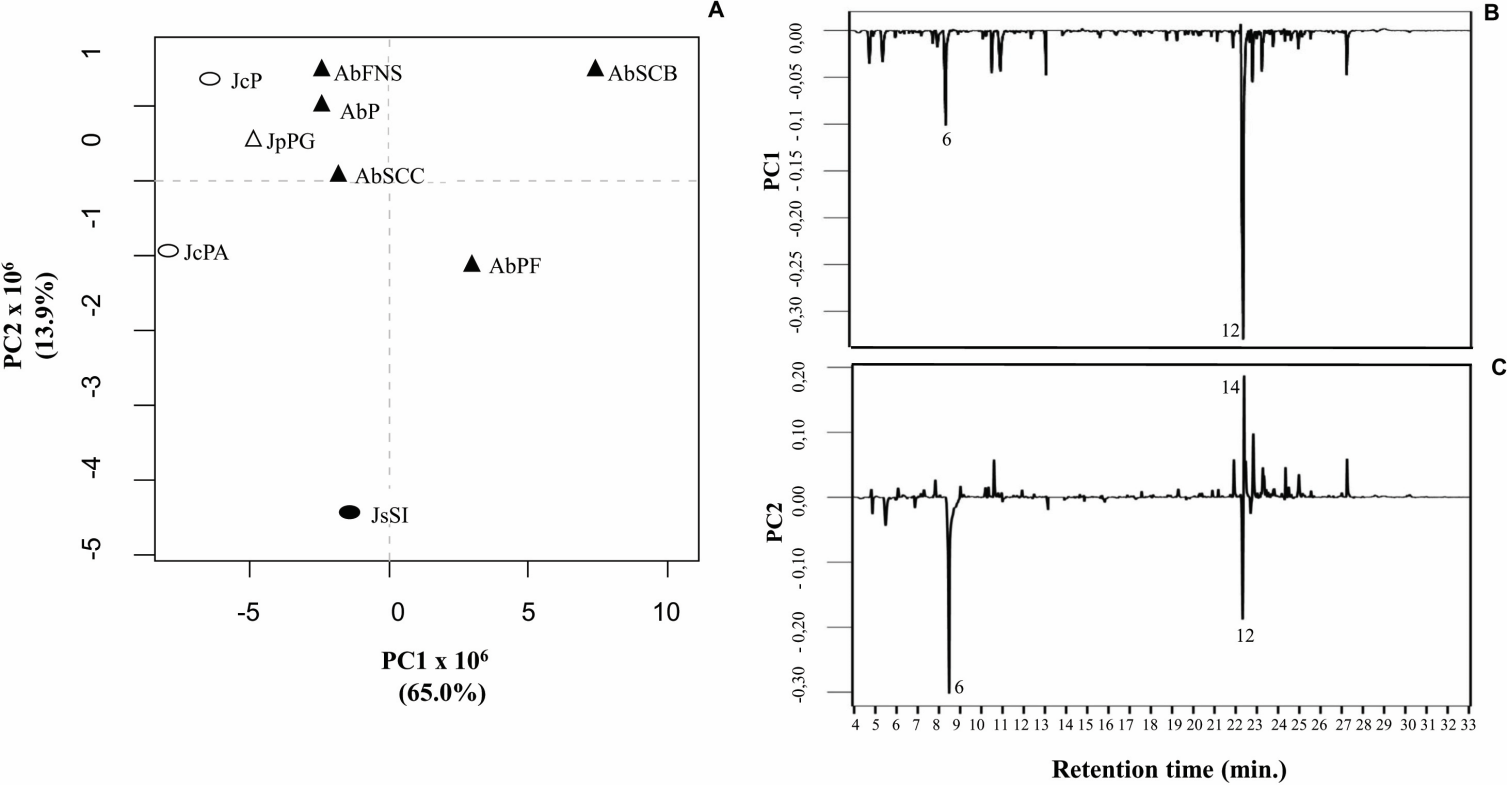
Correlation of the chemical profiles of different GCA species obtained by GC-MS through PCA analysis. (PC1 = 65% and PC2 = 11. 6%). Different symbols represent the diferente species evaluated in this study. AbFNS (*A. beauvoisii*—Fenda de Nossa Senhora); AbP (*A. beauvoisii*—Prainha); AbPF (*A. beauvoisii*—Praia do Forno); AbSCB (*A. beauvoisii*—Saco do Cherne—articuliths bed; AbSCC (*A. beauvoisii*—Saco do Cherne—rocky shore); JcPA (*J. crassa*—Praia dos Anjos); JcP (*J. crassa*—Prainha); JsSI (*J. sagittata*—Saco dos Ingleses) and JpPG (*J. prolifera*—Praia Grande).

We obtained the chemical profiles of nine GCA extracts by GC-MS and used principal component analysis (PCA) to help us assess and explore features of their chemical profiles. The two main components in this study explained 78.9% of the total chromatographic variation (PC1 = 65.0% and PC2 = 13.9%) (Fig. 5A). The PC1 negative axis grouped *A. beauvoisii* (AbFNS, AbP and AbSCC), *J. crassa* (JcP and JcPA), *J. prolifera* (JpPG), and *J. sagittata* (JsSI) extracts. On the other hand, the positive axis grouped *A. beauvoisii* extracts (AbPF and AbSCB), showing their intraspecific chemical variation*.* The compounds responsible for the observed sample distribution were mainly palmitic acid (6) and the sterol (3 *β*)-cholest-5-en-3-ol (12) (Fig. 5B). Meanwhile, the negative axis of PC2 gathered *A. beauvoisii* (AbPF), *J. crassa* (JcPA), and *J. sagittata* (JsSI) extracts. On the other hand, the positive PC2 gathered *A. beauvoisii* (AbFNS, AbP, AbSCC and AbSCB), *J. crassa* (JcP), and *J. prolifera* (JpPG) extracts. In addition to the two compounds already mentioned for PC1, the sterol (3 *β*, 5 *α*)-ergosta-7-en-3-ol (14) (Fig. 5C) was also important in the sample distribution throughout this component, showing intra- and interspecific chemical variability.

### Antibacterial activity

All crude extracts showed antibacterial activity against all five tested bacterial strains. The extracts, when compared with each other, showed significantly different performances in inhibiting all bacterial samples, except when we evaluated them against *V. aestuarianus* (Table 4). In general, comparing extracts within the same GCA species showed no significant differences in their inhibitory responses. Nevertheless, the *A. beauvoisii* extracts collected in Saco do Cherne (AbSCB and AbSCC) were the only ones that avoided this pattern. The AbSCC extract collected in the coastal habitat showed a greater inhibition halo against *P. fluorescens*, *P. irgensii*, and *S. putrefaciens* strains than the AbSCB extract collected on articulith beds. For the other two strains assessed in the test, *P. elyakovii* and *V. aestuarianus*, the extracts showed no significant statistical differences in microbial inhibition. Moreover, as a prominent result, the *J. sagittata* (JsSI) extract differed from the other samples, showing an exceptionally larger inhibition halo than all other extracts when tested against the bacterium *P. elyakovii*.

**Table 4:** Antibacterial activity of extracts after 24 h. ANOVA followed by Tukey’s test. Lowercase letters indicate significant differences between treatments. AbFNS (*A. beauvoisii*—Fenda de Nossa Senhora); AbP (*A. beauvoisii*—Prainha); AbPF (*A. beauvoisii*—Praia do Forno); AbSCB (*A. beauvoisii*—Saco do Cherne—articuliths bed); AbSCC (*A. beauvoisii*—Saco do Cherne—rocky shore); JcPA (*J. crassa*—Praia dos Anjos); JcP (*J. crassa*—Prainha); JsSI (*J. sagittata*—Saco dos Ingleses) e JpPG (*J. prolifera*—Praia Grande), and C+ (positive control—streptomycin).

	**Macroalgae extracts and control**											
**Bacteria species**	AbFNS	AbP	AbPF	AbSCB	AbSCC	JsSI	JcPA	JcP	JpPG	**C+**	*F*	*p*
*Pseudoalteromonas elyakovii*	3.20 ± 0.85^c^	3.57 ± 0.59^c^	3.25 ± 0.62^c^	3.67 ± 0.52^c^	3.25 ± 0.62^c^	4.61 ± 0.46^b^	3.50 ± 0.84^c^	3.14 ± 0.67^c^	2.94 ± 0.55^c^	7.04 ± 0.72^a^	68.32	<0.001
*Pseudomonas fluorescens*	4.16 ± 0.63^b^	3.31 ± 0.36^c^	3.49 ± 0.47^c^	2.90 ± 0.66^c^	4.36 ± 0.45^b^	3.57 ± 0.74^b^	2.82 ± 0.55^c^	3.16 ± 0.78^c^	2.82 ± 0.67^c^	9.46 ± 0.84^a^	150.95	<0.001
*Polaribacter irgensii*	2.58 ± 0.89^c^	3.00 ± 0.45^c^	2.77 ± 0.42^c^	2.87 ± 0.55^c^	4.41 ± 0.53^b^	4.18 ± 0.38^b^	3.16 ± 0.74^c^	3.04 ± 0.39^c^	4.32 ± 0.63^b^	6.05 ± 0.48^a^	77.52	<0.001
*Shewanella putrefaciens*	4.24 ± 0.43^b^	4.20 ± 0.57^b^	2.80 ± 0.87^b^	2.99 ± 0.99^c^	3.93 ± 0.44^b^	4.26 ± 0.40^b^	3.02 ± 0.92^b^	4.12 ± 0.62^b^	3.85 ± 0.66^b^	9.80 ± 0.48^a^	128.62	<0.001
*Vibrio aestuarianus*	3.09 ± 0.41^b^	2.78 ± 0.49^b^	2.54 ± 0.48^b^	2.46 ± 0.52^b^	2.93 ± 0.80^b^	3.15 ± 0.53^b^	2.42 ± 0.42^b^	2.69 ± 0.53^b^	2.43 ± 0.86^b^	6.51 ± 0.52^a^	73.74	<0.001

### Antifouling activity against the mussel *Perna perna*

**Figure 6:**
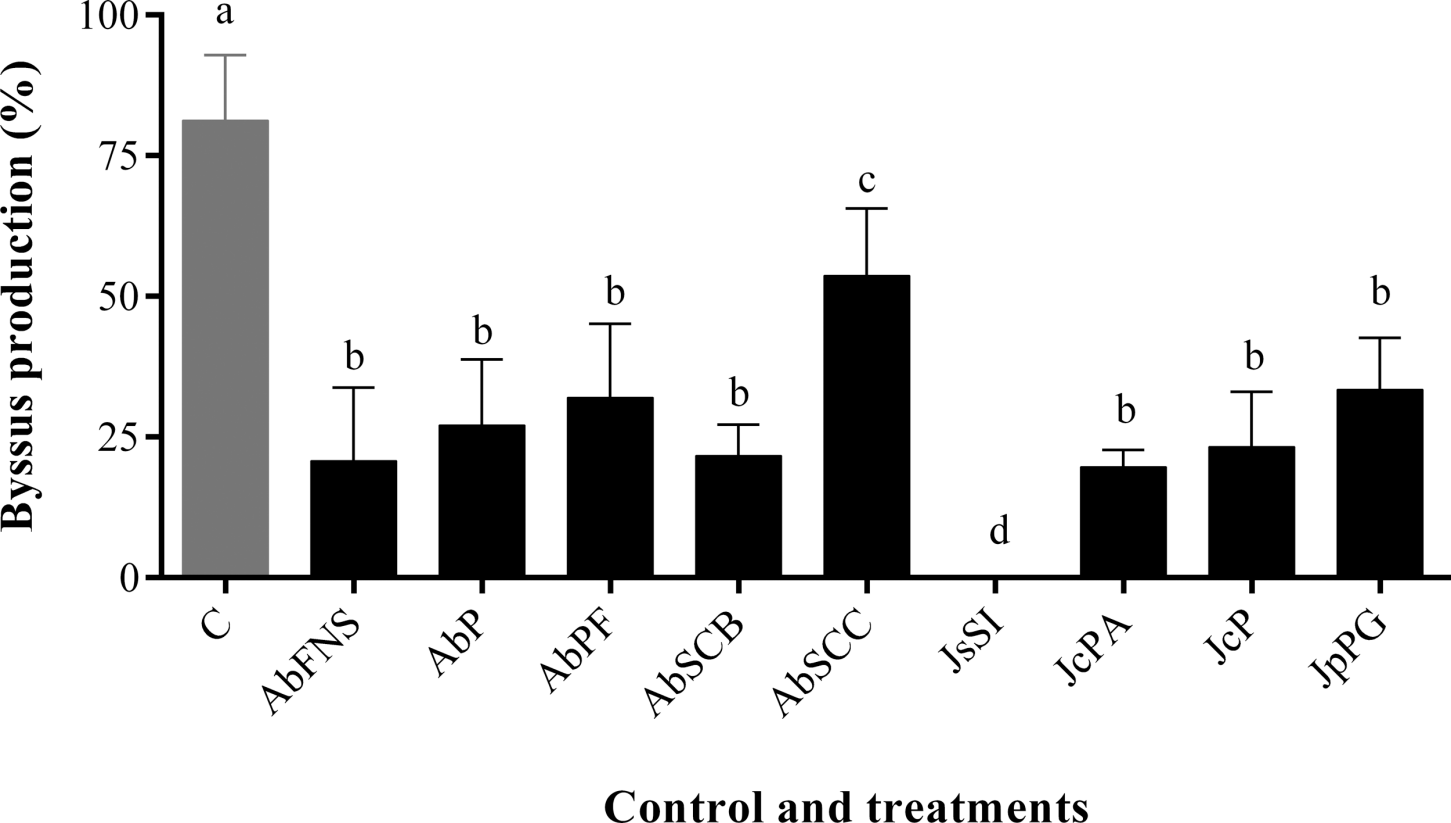
Percentage of byssus production after 24 h. Mean and standard deviation in percentage of byssus production after 24 h. ANOVA followed by Tukey’s test. Lowercase letters indicate significant differences between treatments. AbFNS (*A. beauvoisii*—Fenda de Nossa Senhora); AbP (*A. beauvoisii*—Prainha); AbPF (*A. beauvoisii*—Praia do Forno); AbSCB (*A. beauvoisii*—Saco do Cherne articuliths bed); AbSCC (*A. beauvoisii*—Saco do Cherne—rocky shore); JcPA (*J. crassa*—Praia dos Anjos); JcP (*J. crassa*—Prainha); JsSI (*J. sagittata*—Saco dos Ingleses) and JpPG (*J. prolifera*—Praia Grande), and C (null control—seawater).

**Figure 7:**
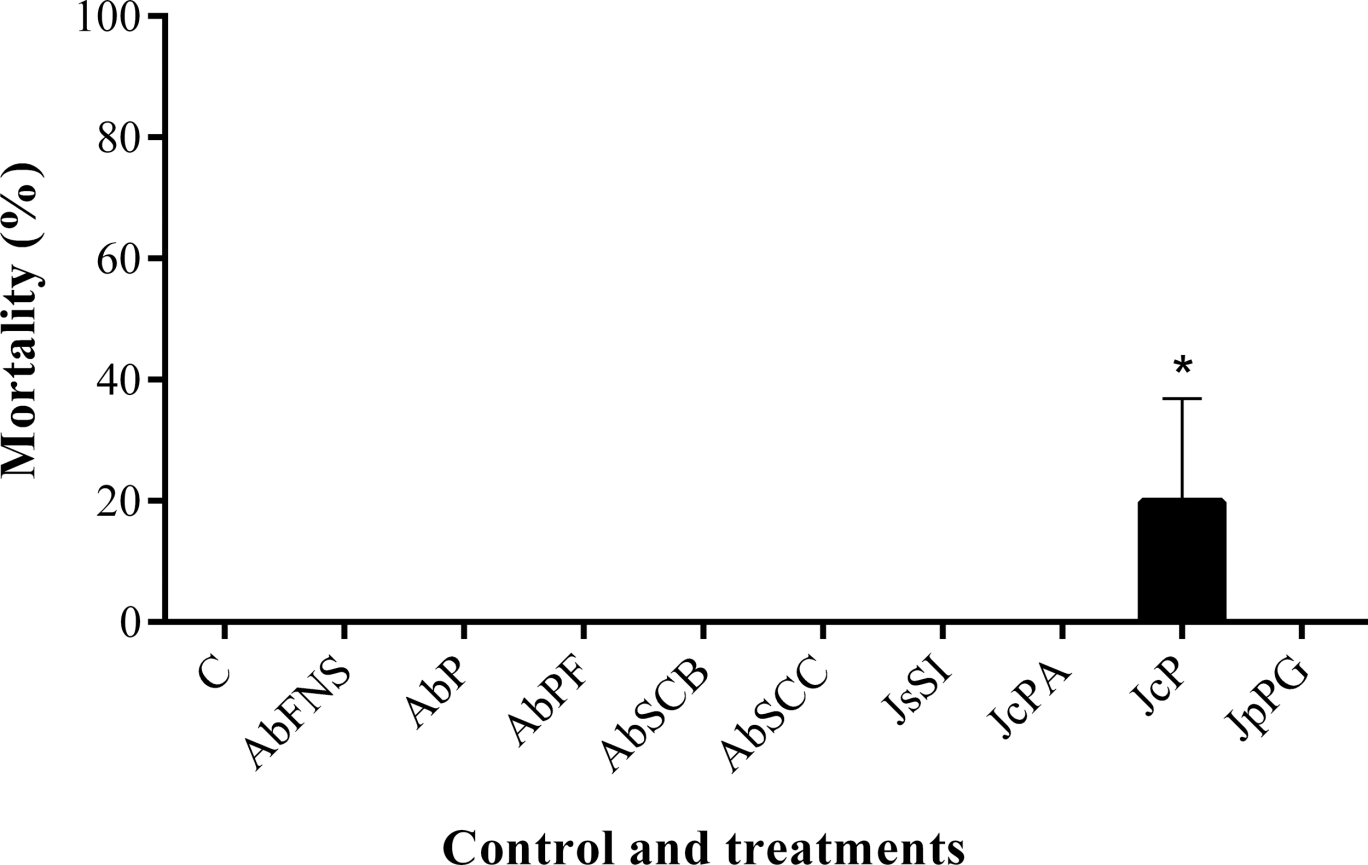
Percentage of mortality after 24 h. Mean and standard deviation in percentage of mortality after 24 h. ANOVA followed by Tukey’s test. Asterisk indicate significant differences between treatments. AbFNS (*A. beauvoisii*—Fenda de Nossa Senhora); AbP (*A. beauvoisii*—Prainha); AbPF (*A. beauvoisii*—Praia do Forno); AbSCB (*A. beauvoisii*—Saco do Cherne—articuliths bed); AbSCC (*A. beauvoisii*—Saco do Cherne—rocky shore); JcPA (*J. crassa*—Praia dos Anjos); JcP (*J. crassa*—Prainha); JsSI (*J. sagittata*—Saco dos Ingleses) and JpPG (*J. prolifera*—Praia Grande), and C (null control—seawater).

All extracts significantly inhibited *P. perna* byssus fixation when compared to the seawater control (ANOVA, *F* = 50.40, *p* < 0.001) (Fig. 6). Similarly, treatment analysis also showed significant differences in their antifouling activities against this mussel. Out of all samples, plates with *J. sagittata* (JsSI) extract showed no byssus fixation and a significantly higher antifouling activity. On the other hand, the AbSCC extract showed the lowest inhibition against the target organism, with a byssus inhibition of 57.18%. Byssus fixation inhibition for all other extracts failed to statistically differ (*p* > 0.05). Regarding the toxicity test, only the JcP extract showed toxic effects against *P. perna*, causing 20% of mussel mortality (ANOVA, *F* = 13.50, *p* < 0.001) (Fig. 7).

## Discussion

In this study, the chromatographic and spectroscopy analyses of the GCA species collected in Arraial do Cabo showed intra- and interspecific variability in their composition, even if obtained from closely located areas with similar oceanographic conditions. For instance, we found certain metabolites in all extracts (*e.g.*, heptadecane, (3 *β*)-cholest-5-en-3-ol, and palmitic acid), with different peak areas and relative abundance (implying a quantitative variation), whereas we only detected compounds such as (Z)-7-hexadecenal in *J. crassa* (JcPA e JcP) and *J. prolifera* (JpPC) species (qualitative variation). On the other hand, pentadecanoic acid was absent in *A. beauvoisii* extracts from Prainha (AbP) and Saco do Cherne (AbSCC and AbSCB) but present in *A. beauvoisii* from Fenda de Nossa Senhora (AbFNS) and Praia do Forno (AbPF), reinforcing a potential intraspecific qualitative variability.

We found different chemical classes in GCA extracts according to GC-MS analysis, followed by their tentative identification on NIST and GNPS databases. Fatty acids, hydrocarbons, sterols, and fatty alcohols were recurrent classes of metabolites in this study. The analysis by molecular networking we performed on the GNPS platform was an important metabolomic tool, enabling us to observe groupings of molecular families consisting of compounds belonging to the same chemical class, which contributed to the identification of compounds on the NIST library.

Marine macroalgae can synthesize an extensive number of compounds with a wide structural and functional diversity (Biris-Dorhoi et al., 2020). Several environmental factors, such as temperature (Sudatti et al., 2011), nutrient availability (Stengel, Connan & Popper, 2011), and different ecological interactions (Stengel, Connan & Popper, 2011) in the environment are known to influence the production of these compounds. The Arraial do Cabo maritime area of shows two distinct morphological features, in which its inner portion (Arraial do Cabo Bay) is characterized as a sheltered zone with warmer waters, whereas its outer part suffers strong influence of wave action and seasonal upwelling. The occurrence of this phenomenon decreases water temperatures (<18  °C) and increases the availability of nutrients in this external part (Guimaraens & Coutinho, 1996; Candella, 2009; Batista, Granthom-Costa & Coutinho, 2020). Although we obtained our GCA samples from both internal (sheltered) and external (exposed) areas in Arraial do Cabo during the summertime—when a stronger influence of the upwelling system is expected—, we observed quantitative and qualitative variability in the chemical profile of the extracts between the samples collected from areas with similar environmental conditions, rather than only when we compared samples from both contrasting environments.

The sterol (3 *β*)-cholest-5-en-3-ol was the most abundant compound identified in the databases (GNPS and NIST), present in almost all extracts and ranging from 12.63% to 39.07% of their peak area, with the exception of *Jania sagittata* (JsSI). This compound has also been reported as the most abundant compound in methanolic extracts of *Jania rubens* (Linnaeus) J.V. Lamouroux (24.25%) collected in Egypt (Ahmed et al., 2011). On the other hand, in the extract (methanol and hexane, 1:1 v/v) of *Amphiroa anceps* (Lamarck) Decaisne, (3 *β*)-cholest-5-en-3-ol failed to feature as one of the major compounds, showing only 2% of peak area (Mofeed et al., 2022). This class of metabolite is essential to the cellular structure of several organisms and it is associated with different biological activities, such as antioxidant, antiviral, and antitumor activity (Alassali et al., 2016; Thirumurugan et al., 2018; Fagundes & Wagner, 2021).

Palmitic acid, also found in all extracts, showed a higher abundance in *J. sagittata* extracts (JsSI) than in other samples (31.35% of peak area). Venkatesalu et al. (2012) analyzed the composition of fatty acids in different species of marine macroalgae, including geniculate calcareous algae. Their results showed a greater abundance of palmitic acid in *Jania spectabilis* (Harvey ex Grunow) J.H. Kim, Guiry & H.G. Choi (formerly *Cheilosporum spectabile* Harvey ex Grunow) (11.72%), *Amphiroa foliacea* J.V. Lamouroux (91.56%), and *Amphiroa* sp. (92.92%) collected in spring, during and after a monsoon, respectively. Palmitic acid has also been identified as the most abundant compound in *A. anceps* - 57.57% (Jayasree et al., 2012) and *J. rubens* - 34.22% (Caf et al., 2019). Studies with fatty acids showing their potential biological activity have increasingly become more attractive. Antibacterial (Desbois & Smith, 2010; Casillas-Vargas et al., 2021), antioxidant (Henry et al., 2002), antifungal (Guimarães & Venâncio, 2022), and antifouling activity (Goto et al., 1992; Gao et al., 2014) are some of the reported biotechnological activities in this class of metabolite.

We also observed biological activity variability among GCA extracts against the five chosen bacterial strains. Assays often use marine bacteria to assess antibiofilm activity, as the inhibition of specific species can directly affect the colonization of fouling organisms (Dobretsov, Teplitski & Paul, 2009; Da Gama, Plouguerné & Pereira, 2014). The test performed against bacterial strains enabled us to find significant differences in *A. beauvoisii* extracts. The sample collected on the rocky shore of Saco do Cherne (AbSCC) provided better inhibitory results, whereas the material sampled at the same location but from a different habitat (articulith beds at Saco do Cherne, AbSCB) showed a weaker response. The different inhibitory performances of *A. beauvoisii* extracts can be reasonably associated with their chemical composition, especially considering the habitat of the samples. The intertidal rocky shore habitat is susceptible to daily stress with variations in irradiance, temperature, desiccation, and water movement (Fields et al., 1993; Helmuth, 1999; Helmuth, 2002; Massa et al., 2009). On the other hand, the articulith beds at Saco do Cherne (15–18 m depths) show a more stable habitat with fewer environmental changes (Tâmega et al., 2021). Deepa, Srikumar & Padmakumar, 2014 also showed the inhibitory activity of the ethanolic extract of *A. anceps* against marine bacteria of the genus *Vibrio*. Studies have also evaluated the genus *Amphiroa* against pathogenic microorganisms. Vlashos, Critchley & Holy (1997) obtained results for the activity of ethanolic extracts of *Amphiroa ephedraea* (Lamarck) Decaisne against four species of fungi and 11 species of bacteria, more effectively inhibiting *Bacillus subtilis* EL39 (20–25 mm). On the other hand, Ballesteros, Martín & Uriz (1992) found inactivity or low inhibition for extracts of *A. beauvoisii, Amphiroa cryptarthrodia* Zanardini, and *Amphiroa rigida* J.V. Lamouroux against different biological models (bacteria, viruses and fungi). Extracts of *A. anceps* (MeOH/Hex 1:1) containing 1,2-benzenedicarboxylic acid, diisooctyl ester (30.4%), and pentadecanoic acid, 14-methyl-, and methyl ester (29.5%) as their most abundant compounds showed antibacterial activity against *Salmonella typhimurium, Staphylococcus aureus*, and *Escherichia coli* and antiviral activity against rotavirus and coxsackievirus B3 (Mofeed et al., 2022).

This study, considering the extracts of *J. crassa,* also found its inhibitory activity against strains of marine bacteria. However, results showed no significant statistical differences between them (JcP and JcPA), except against the bacterium *S. putrefaciens*, in which the sample collected at Prainha (JcP) was more effective than the one obtained at Praia dos Anjos (JcPA). This observation, according to the results of our chemical profile, showed intraspecific variation in the chemical composition of these samples. The literature also reports the biological potential of the genus *Jania* by making use of other models of pathogenic microorganisms. Ethanolic extracts of *Jania pedunculata* var. *adhaerens* (J.V. Lamouroux) A.S. Harvey and Woelkerling & Revier (formerly *Jania adhaerens* J.V. Lamouroux) showed a mild activity against four strains of marine bacteria, including the genus *Pseudomonas* (also evaluated in this work) with an inhibition zone of 0.5 mm (Kantida, Asha & Sujatha, 2012). In the study conducted by Sasikala & Geetha (2017), the methanolic extract of the species *J. rubens* was one of the most active against different bacterial strains, especially against *Enterococcus faecalis* and *Streptococcus pyogenes*. Specifically regarding the *J. crassa* species, only Soares et al. (2012) reported the inhibitory action of its extract (dichloromethane/methanol 1:1) against two types of herpes viruses.

The *J. sagittata* (JsSI) extract showed one of the most satisfactory inhibitory responses against four species of marine bacteria (*P. elyakovii, P. fluorescens, P. irgensii*, and *S. putrefaciens*). Its chemical composition, containing palmitic acid as the most abundant compound, may be associated with this activity. The methanolic extract of *J. spectabilis* (formerly *Cheilosporum spectabile*) also showed activity against two species of diatoms *Dickieia subinflata* (formerly *Navicula subinflata*) and *Nitzschia palea* (Deepa, Srikumar & Padmakumar, 2014). Vlashos, Critchley & Holy (1997) observed that the extract of *J. sagittata* inhibited the growth of four species of fungi and 12 species of bacteria, having the most remarkable responses against *Bacillus subtilis* EL39*, Micrococcus* sp., and *Staphylococcus aureus.* The study by Stirk et al. (2003) evaluated the activity of the ethanolic extract of the genus *Jania* against strains of Gram-positive and Gram-negative bacteria. Studies on the biotechnological potential of this genus are scarcer. Thus, this study offers another promising alternative for the use of natural products from macroalgae with antibacterial potential.

Moreover, antifouling experiments using *P. perna* have shown this mollusk as an excellent model organism for fouling studies due to its fast and clear response to bioactive compounds (Da Gama et al., 2003; Barbosa et al., 2007; Da Gama et al., 2008; Plouguerné et al., 2010). Marine fouling organisms use their byssal threads to firmly attach themselves to various submerged structures (such as ship hulls), causing serious economic problems for the shipbuilding industry (Wang et al., 2016). In this study, the crude extracts obtained from geniculate calcareous algae inhibited byssus production, among which that of *J. sagittata* (JsSI) showed the highest levels of biological activity. *J. crassa* collected at Prainha (JcP) was the only extract that showed considerable toxic effects on the target organism, with a 20% mortality. In a similar experiment, Medeiros, Gama & Gallerani (2007) reported the antifouling potential against the mussel *P. perna* of the crude extract of four species of macroalgae, including one species of *Jania*. *J. rubens* constituted one of the most active species against the mollusk but, in contrast, caused no mortality.

## Conclusions

The results obtained for the *A. beauvoisii*, *J. sagittata, J. crassa*, and *J. prolifera* extracts collected at Arraial do Cabo are unprecedented regarding their chemical composition, intra- and interspecific chemical variability, and activity against the tested models. Moreover, this study contributed new information about the chemical compounds this group of seaweeds produces and showed its antifouling potential. These GCA species may be the subject of future studies to obtain new bioactive compounds with potential applications in maritime areas.

##  Supplemental Information

10.7717/peerj.15731/supp-1 Supplemental Information 1 Raw Data: Toxicity mussel

10.7717/peerj.15731/supp-2 Supplemental Information 2 Raw Data: bacteria

10.7717/peerj.15731/supp-3 Supplemental Information 3 Raw Data: Fixed byssus

## Additional Information and Declarations

### Data Availability

The following information was supplied regarding data availability: The raw data are available in the Supplemental Files.

## References

[ref-1] Ahmed HH, Hegazi MM, Abd-Alla HI, Eskander EF, Ellithey MS (2011). Antitumour and antioxidant activity of some red sea seaweeds in Ehrlich ascites carcinoma *in vivo*. Zeitschrift für Naturforschung.

[ref-2] Akbary P, Adeshina I, Jahanbakhshi A (2020). Growth performance, digestive enzymes, antioxidant activity and immune responses of *Litopenaeus vannamei* fed with *Jania adhaerens* J.V. Supplemented diet against *Photobacterium damselae* infection. Animal Feed Science and Technology.

[ref-3] Alassali A, Cybulska I, Brudecki GP, Farzanah R, Thomsen MH (2016). Methods for upstream extraction and chemical characterization of secondary metabolites from algae biomass. Advanced Techniques in Biology & Medicine.

[ref-4] Ali A, Jamil MI, Jiang J, Shoaib M, Amin BU, Luo S, Zhan X, Chen F, Zhang Q (2020). An overview of controlled-biocide-release coating based on polymer resin for marine antifouling applications. Journal of Polymer Research.

[ref-5] Ballesteros E, Martín D, Uriz MJ (1992). Biological activity of extracts from some Mediterranean macrophytes. Botanica Marina.

[ref-6] Barbosa JP, Fleury BG, da Gama BAP, Teixeira VL, Pereira RC (2007). Natural products as antifoulants in the Brazilian brown alga *Dictyota pfaffii* (Phaeophyta, Dictyotales). Biochemical Systematics and Ecology.

[ref-7] Batista D, Granthom-Costa LV, Coutinho R (2020). Biodiversidade marinha dos costões rochosos de Arraial do Cabo: histórico, ecologia e conservação.

[ref-8] Batista-Andrade JA, Caldas SS, Batista RM, Castro IB, Fillmann G, Primel EG (2018). From TBT to booster biocides: levels and impacts of antifouling along coastal areas of Panama. Environmental Pollution.

[ref-9] Belem AL, Castelao RM, Albuquerque ALS (2013). Controls of subsurface temperature variability in a western boundary upwelling system. Geophysical Research Letters.

[ref-10] Biris-Dorhoi E-S, Michiu D, Pop CR, Rotar AM, Tofana M, Pop OL, Socaci SA, Farcas AC (2020). Macroalgae—a sustainable source of chemical compounds with biological activities. Nutrients.

[ref-11] Caf F, Özdemir NŞen, Yılmaz Ö, Durucan F, Ak I (2019). Fatty acid and lipophilic vitamin composition of seaweeds from Antalya and Çanakkale (Turkey). Grasas y Aceites.

[ref-12] Candella RN (2009). Meteorologically induced strong seiches observed at Arraial do Cabo, RJ, Brazil. Physics and Chemistry of the Earth.

[ref-13] Cao S, Wang J, Chen H, Chen D (2011). Progress of marine biofouling and antifouling technologies. Chinese Science Bulletin.

[ref-14] Carvalho AP, Batista D, Dobretsov S, Coutinho R (2016). Extracts of seaweeds as potential inhibitors of quorum sensing and bacterial growth. Journal of Applied Phycology.

[ref-15] Casillas-Vargas G, Ocasio-Malavé C, Medina S, Morales-Guzmán C, Del Valle RG, Carballeira NM, Sanabria-Ríos DJ (2021). Antibacterial fatty acids: an update of possible mechanisms of action and implications in the development of the next-generation of antibacterial agents. Progress in Lipid Research.

[ref-16] Castelão RM (2012). Sea surface temperature and wind stress curl variability near a cape. Journal of Physical Oceanography.

[ref-17] Cikoš A-M, Flanjak I, Bojanić K, Babić S, Čižmek L, Čož Rakovac R, Jokić S, Jerković I (2021). Bioprospecting of coralline red alga *Amphiroa rigida* J.V. Lamouroux: volatiles, fatty acids and pigments. Molecules.

[ref-18] Da Gama BAP, Carvalho AGV, Weidner K, Soares AR, Coutinho R, Fleury BG, Teixeira VL, Pereira RC (2008). Antifouling activity of natural products from Brazilian seaweeds. Botanica Marina.

[ref-19] Da Gama BAP, Pereira RC, Soares AR, Teixeira VL, Yoneshigue-Valentin Y (2003). Is the mussel test a good indicator of antifouling activity? A comparison between laboratory and field assays. Biofouling.

[ref-20] Da Gama BAP, Plouguerné E, Pereira RC, Bourgougnon N, Jacquot J-P, Gadal P (2014). The antifouling defence mechanisms of marine macroalgae. Advances in botanical research: sea plants.

[ref-21] Dang H, Li T, Chen M, Huang G (2007). Cross-ocean distribution of Rhodobacterales bacteria as primary surface colonizers in temperate coastal marine waters. Applied and Environmental Microbiology.

[ref-22] Davidson I, Scianni C, Hewitt C, Everett R, Holm E, Tamburri M, Ruiz G (2016). Mini-review: assessing the drivers of ship biofouling management –aligning industry and biosecurity goals. Biofouling.

[ref-23] Deepa S, Srikumar M, Padmakumar KP (2014). Antifouling potential of selected macroalgae from the Gulf of Mannar, India. International Journal of Bioassays.

[ref-24] Desbois AP, Smith VJ (2010). Antibacterial free fatty acids: activities, mechanisms of action and biotechnological potential. Applied Microbiology and Biotechnology.

[ref-25] Devi P, Wahidulla S, Kamat T, D’Souza L (2011). Screening marine organisms for antimicrobial activity against clinical pathogens. Indian Journal of Geo-Marine Sciences.

[ref-26] Dobretsov S, Teplitski M, Paul V (2009). Mini-review: quorum sensing in the marine environment and its relationship to biofouling. Biofouling.

[ref-27] Fagundes MB, Wagner R (2021). Sterols biosynthesis in algae. Bioactive compounds—biosynthesis, characterization and applications.

[ref-28] Ferreira O, Rijo P, Gomes JF, Santos R, Monteiro S, Vilas-Boas C, Correia-da Silva M, Almada S, Alves LG, Bordado JC, Silva ER (2020). Biofouling inhibition with grafted econea biocide: toward a nonreleasing eco-friendly multiresistant antifouling coating. ACS Sustainable Chemistry & Engineering.

[ref-29] Fields PA, Graham JB, Rosenblatt RH, Somero GN (1993). Effects of expected global climate change on marine faunas. Trends in Ecology and Evolution.

[ref-30] Foster MS (2001). Rhodoliths: between rocks and soft places. Journal of Phycology.

[ref-31] Gao M, Li F, Su R, Wang K, Li X, Lu W (2014). Antifouling potential of the marine microalga *Dunaliella salina*. World Journal of Microbiology and Biotechnology.

[ref-32] Goto R, Kado R, Muramoto K, Kamiya H (1992). Fatty acids as antifoulants in a marine sponge. Biofouling.

[ref-33] Guimarães A, Venâncio A (2022). The potential of fatty acids and their derivatives as antifungal agents: a review. Toxins.

[ref-34] Guimaraens MA, Coutinho R (1996). Spatial and temporal variation of benthic marine algae at the Cabo Frio upwelling region, Rio de Janeiro, Brazil. Aquatic Botany.

[ref-35] Han X, Wu J, Zhang X, Shi J, Wei J, Yang Y, Wu B, Feng Y (2021). Special issue on advanced corrosion-resistance materials and emerging applications. The progress on antifouling organic coating: from biocide to biomimetic surface. Journal of Materials Science & Technology.

[ref-36] Harvey A, Woelkerling W, Farr T, Neill K, Nelson W (2005). Coralline algae of central New Zealand.

[ref-37] Helmuth B (1999). Thermal biology of rocky intertidal mussels: quantifying body temperatures using climatological data. Ecology.

[ref-38] Helmuth B (2002). How do we measure the environment? Linking intertidal thermal physiology and ecology through biophysics. Integrative and Comparative Biology.

[ref-39] Henry GE, Momin RA, Nair MG, Dewitt DL (2002). Antioxidant and cyclooxygenase activities of fatty acids found in food. Journal of Agricultural and Food Chemistry.

[ref-40] Jayasree NB, Aneesh TP, Prabhakar V, Anandan R (2012). GC-MS, HPLC and AAS analysis of fatty acids, amino acids and minerals in red alga *Amphiroa anceps*. International Journal of Phatmaceutical Sciences.

[ref-41] Johansen HW (1981). Coralline algae, a first synthesis.

[ref-42] Kantida SR, Asha KRT, Sujatha S (2012). Influence of bioactive compounds from seaweeds and its biocidal and corrosion inhibitory effect on mild steel. Research Journal of Environmental Toxicology.

[ref-43] Laborel JL (1970). Madréporaires et hydrocoralliaires récifaux des cotes brésiliennes: systématique, écologie, répartition verticale et géographique. Annales de l’Institut Océanographique.

[ref-44] Lanari MO, Coutinho R (2014). Reciprocal causality between marine macroalgal diversity and productivity in an upwelling area. Oikos.

[ref-45] Mansur AA, Brown MT, Billington RA (2020). The cytotoxic activity of extracts of the brown alga *Cystoseira tamariscifolia* (Hudson) Papenfuss, against cancer cell lines changes seasonally. Journal of Applied Phycology.

[ref-46] Maréchal J, Hellio C (2009). Challenges for the development of new non-toxic antifouling solutions. International Journal of Molecular Sciences.

[ref-47] Martín-Rodríguez AJ, Babarro JMF, Lahoz F, Sansón M, Martín VS, Norte M, Fernández JJ (2015). From broad-spectrum biocides to quorum sensing disruptors and mussel repellents: antifouling profile of alkyl triphenylphosphonium salts. PLOS ONE.

[ref-48] Massa SI, Arnaud-Haond S, Pearson GA, Serrao EA (2009). Temperature tolerance and survival of intertidal populations of the seagrass *Zostera noltii* (Hornemann) in Southern Europe (Ria Formosa, Portugal). Hydrobiologia.

[ref-49] Medeiros HE, Gama BAP, Gallerani G (2007). Antifouling activity of seaweed extracts from Guarujá, São Paulo, Brazil. Brazilian Journal of Oceanography.

[ref-50] Mofeed J, Deyab M, Mohamed A, Moustafa M, Negm S, El-Bilawy E (2022). Antimicrobial activities of three seaweeds extract against some human viral and bacterial pathogens. Biocell.

[ref-51] Nash MC, Adey W (2017). Multiple phases of mg-calcite in crustose coralline algae suggest caution for temperature proxy and ocean acidification assessment: lessons from the ultrastructure and biomineralization in *Phymatolithon* (Rhodophyta, Corallinales). Journal of Phycology.

[ref-52] Nielsen N-PV, Carstensen JM, Smedsgaard J (1998). Aligning of single and multiple wavelength chromatographic profiles for chemometric data analysis using correlation optimised warping. Journal of Chromatography A.

[ref-53] Othmani A, Bunet R, Bonnefont J-L, Briand J-F, Culioli G (2015). Settlement inhibition of marine biofilm bacteria and barnacle larvae by compounds isolated from the Mediterranean brown alga *Taonia atomaria*. Journal of Applied Phycology.

[ref-54] Plouguerné E, Hellio C, Cesconetto C, Thabard M, Mason K, Véron B, Pereira RC, da Gama BAP (2010). Antifouling activity as a function of population variation in *Sargassum vulgare* from the littoral of Rio de Janeiro (Brazil). Journal of Applied Phycology.

[ref-55] Qian P-Y, Li Z, Xu Y, Li Y, Fusetani N (2015). Mini-review: marine natural products and their synthetic analogs as antifouling compounds: 2009–2014. Biofouling.

[ref-56] Raj T, Muthukumar A, Vignesh M, Charumathi M, Suji H (2019). Efficacy of seaweed extract against downy mildew of grapes caused by *Plasmopara viticola*. Plant archives.

[ref-57] Righini H, Francioso O, Di Foggia M, Prodi A, Quintana AM, Roberti R (2021). Tomato seed biopriming with water extracts from *Anabaena minutissima*, Ecklonia maxima and *Jania adhaerens* as a new agro-ecological option against *Rhizoctonia solani*. Scientia Horticulturae.

[ref-58] Riosmena-Rodríguez R, Nelson W, Aguirre J (2017). Rhodolith/mäerl beds: a global perspective.

[ref-59] Sánchez-Lozano I, Hernández-Guerrero CJ, Muñoz Ochoa M, Hellio C (2019). Biomimetic approaches for the development of new antifouling solutions: study of incorporation of macroalgae and sponge extracts for the development of new environmentally-friendly coatings. International Journal of Molecular Sciences.

[ref-60] Sasikala C, Geetha R (2017). Comparative study on antimicrobial activity of seaweeds. Asian Journal of Pharmaceutical and Clinical Research.

[ref-61] Silva ER, Ferreira O, Ramalho PA, Azevedo NF, Bayón R, Igartua A, Bordado JC, Calhorda MJ (2018). Eco-friendly non-biocide-release coatings for marine biofouling prevention. Science of the Total Environment.

[ref-62] Soares AR, Robaina MCS, Mendes GS, Silva TSL, Gestinari LMS, Pamplona OS, Yoneshigue-Valentin Y, Kaiser CR, Romanos MTV (2012). Antiviral activity of extracts from Brazilian seaweeds against Herpes simplex virus. Revista Brasileira de Farmacognosia.

[ref-63] Stengel DB, Connan S, Popper ZA (2011). Algal chemodiversity and bioactivity: sources of natural variability and implications for commercial application. Biotechnology Advances.

[ref-64] Stirk WA, Schwalb AN, Light ME, Medková J, Lenobel R, Strnad M, van Staden J (2003). Potential medicinal value of some South African seaweeds. South African Journal of Botany.

[ref-65] Sudatti DB, Fujii MT, Rodrigues SV, Turra A, Pereira RC (2011). Effects of abiotic factors on growth and chemical defenses in cultivated clones of *Laurencia dendroidea* J. Agardh (Ceramiales, Rhodophyta). Marine Biology.

[ref-66] Tâmega FTS, Perna GHH, Spotorno-Oliveira P, Riosmena-Rodriguez R, Gonçalves JEA (2017). A unique free-living geniculate coralline algal bed formation. Marine Biodiversity.

[ref-67] Tâmega FTS, Torrano-Silva BN, Oliveira MC, Spotorno-Oliveira P, Calazans SH, Rosas-Alquicira EF, Coutinho R, Peña V (2021). Identification of ‘articuliths’ in a unique algal bed formation from Brazil and description of *Jania cabista* sp. nov. (Corallinales, Rhodophyta). Phycologia.

[ref-68] Thirumurugan D, Cholarajan A, Raja SSS, Vijayakumar R (2018). An introductory chapter: secondary metabolites. Secondary metabolites - sources and applications.

[ref-69] Venkatesalu V, Sundaramoorthy P, Anantharaj M, Chandrasekaran M, Senthilkumar A (2012). Seasonal variation on fatty acid composition of some marine macro algae from Gulf of Mannar. Marine Biosphere Reserve, Southeast cost of India, Indian Journal of Geo-Marine Sciences.

[ref-70] Vimala R (2016). Marine organisms: a potential source of natural antifouling metabolites. International Journal of ChemTech Research.

[ref-71] Vlashos V, Critchley AT, Holy Avon (1997). Antimicrobial activity of extracts from selected southern African marine macroalgae. South African Journal of Science.

[ref-72] Wahl M (1989). Marine epibiosis. I. Fouling and antifouling: some basic aspects. Marine Ecology Progress Series.

[ref-73] Wang H, Li Y, Huang H, Xu X, Wang Y (2011). Toxicity evaluation of single and mixed antifouling biocides using the *Strongylocentrotus intermedius* sea urchin embryo test. Environmental Toxicology and Chemistry.

[ref-74] Wang M, Carver JJ, Phelan VV, Sanchez LM, Garg N, Peng Y, Nguyen DD, Watrous J, Kapono CA, Luzzatto-Knaan T, Porto C, Bouslimani A, Melnik AV, Meehan MJ, Liu WT, Crüsemann M, Boudreau PD, Esquenazi E, Sandoval-Calderón M, Kersten RD, Pace LA, Quinn RA, Duncan KR, Hsu CC, Floros DJ, Gavilan RG, Kleigrewe K, Northen T, Dutton RJ, Parrot D, Carlson EE, Aigle B, Michelsen CF, Jelsbak L, Sohlenkamp C, Pevzner P, Edlund A, McLean J, Piel J, Murphy BT, Gerwick L, Liaw CC, Yang YL, Humpf HU, Maansson M, Keyzers RA, Sims AC, Johnson AR, Sidebottom AM, Sedio BE, Klitgaard A, Larson CB, Cab P, Torres-Mendoza D, Gonzalez DJ, Silva DB, Marques LM, Demarque DP, Pociute E, O’Neill EC, Briand E, Helfrich EJN, Granatosky EA, Glukhov E, Ryffel F, Houson H, Mohimani H, Kharbush JJ, Zeng Y, Vorholt JA, Kurita KL, Charusanti P, McPhail KL, Nielsen KF, Vuong L, Elfeki M, Traxler MF, Engene N, Koyama N, Vining OB, Baric R, Silva RR, Mascuch SJ, Tomasi S, Jenkins S, Macherla V, Hoffman T, Agarwal V, Williams PG, Dai J, Neupane R, Gurr J, Rodríguez AMC, Lamsa A, Zhang C, Dorrestein K, Duggan BM, Almaliti J, Allard PM, Phapale P, Nothias LF, Alexandrov T, Litaudon M, Wolfender JL, Kyle JE, Metz TO, Peryea T, Nguyen DT, Van Leer D, Shinn P, Jadhav A, Müller R, Waters KM, Shi W, Liu X, Zhang L, Knight R, Jensen PR, Palsson BO, Pogliano K, Linington RG, Gutiérrez M, Lopes NP, Gerwick WH, Moore BS, Dorrestein PC, Bandeira N (2016). Nature Biotechnology.

[ref-75] Wehrens R (2011). Chemometrics with R.

